# *Pseudomonas aeruginosa* Exotoxin Y-Mediated Tau Hyperphosphorylation Impairs Microtubule Assembly in Pulmonary Microvascular Endothelial Cells

**DOI:** 10.1371/journal.pone.0074343

**Published:** 2013-09-04

**Authors:** Ron Balczon, Nutan Prasain, Cristhiaan Ochoa, Jason Prater, Bing Zhu, Mikhail Alexeyev, Sarah Sayner, Dara W. Frank, Troy Stevens

**Affiliations:** 1 Department of Cell Biology and Neuroscience, University of South Alabama, Mobile, Alabama, United States of America; 2 Center for Lung Biology, University of South Alabama, Mobile, Alabama, United States of America; 3 Department of Pediatrics, University of Indiana School of Medicine, Indianapolis, Indiana, United States of America; 4 Department of Pharmacology, University of South Alabama, Mobile, Alabama, United States of America; 5 Department of Medicine, University of South Alabama, Mobile, Alabama, United States of America; 6 Department of Microbiology and Molecular Genetics, Medical College of Wisconsin, Milwaukee, Wisconsin, United States of America; University of Kentucky, United States of America

## Abstract

*Pseudomonas aeruginosa* uses a type III secretion system to introduce the adenylyl and guanylyl cyclase exotoxin Y (ExoY) into the cytoplasm of endothelial cells. ExoY induces Tau hyperphosphorylation and insolubility, microtubule breakdown, barrier disruption and edema, although the mechanism(s) responsible for microtubule breakdown remain poorly understood. Here we investigated both microtubule behavior and centrosome activity to test the hypothesis that ExoY disrupts microtubule dynamics. Fluorescence microscopy determined that infected pulmonary microvascular endothelial cells contained fewer microtubules than control cells, and further studies demonstrated that the microtubule-associated protein Tau was hyperphosphorylated following infection and dissociated from microtubules. Disassembly/reassembly studies determined that microtubule assembly was disrupted in infected cells, with no detectable effects on either microtubule disassembly or microtubule nucleation by centrosomes. This effect of ExoY on microtubules was abolished when the cAMP-dependent kinase phosphorylation site (Ser-214) on Tau was mutated to a non-phosphorylatable form. These studies identify Tau in microvascular endothelial cells as the target of ExoY in control of microtubule architecture following pulmonary infection by *Pseudomonas aeruginosa* and demonstrate that phosphorylation of tau following infection decreases microtubule assembly.

## Introduction

Cell shape is controlled by interactions between microfilaments, intermediate filaments and microtubules. In endothelial cells, a rim of cortical microfilaments stabilizes junctional complexes responsible for cell-cell and cell-matrix adhesion processes. Moreover, microfilaments generate an inward tension that is opposed by microtubules extending toward the cell membrane to form a tightly adherent monolayer of endothelial cells that allows regulated movement of water, solutes, macromolecules and cells between the blood and the underlying tissues [1], [2]. Injury or agents that disrupt the endothelial cytoskeleton lead to decreased adhesion resulting in the formation of gaps between endothelial cells, and subsequently tissue edema [3].

cAMP is a secondary messenger that controls integrity of the endothelial cell barrier. Various signaling ligands lead to the production of a membrane localized cAMP pool, which is responsible for stabilizing the membrane-associated actin cytoskeleton resulting in strengthening of cell adhesion processes [4]–[8]. Although cAMP is capable of diffusion within the cytosol, multiple mechanisms are utilized for maintaining elevated levels of cAMP near the membrane relative to levels deeper in the cell, including anchoring adenylyl cyclases to the plasma membrane [7], [9], steric inhibition of diffusion by intracellular membranes [10]–[13], and localization of phosphodiesterases to the cortical area of cells [14], [15]. Proper maintenance of this regional cAMP pool is critical for maintaining strength of the endothelial barrier. The essential nature of cAMP for barrier integrity can be seen by the action of inflammatory agonists, such as thrombin and bradykinin; these inflammatory agents activate signal transduction events that result in decreased levels of membrane-associated cAMP, resulting in weakened cell adhesion, the formation of gaps between neighboring endothelial cells, and tissue edema [2], [5], [16].

Whereas membrane-associated cAMP pools strengthen endothelial cell adhesion, activation of soluble adenylyl cyclases that elevate cytosolic cAMP decrease cell adhesion forming inter-endothelial cell gaps, responses demonstrated experimentally by Sayner et al. [7], [17], [18] and Prasain et al. [19]. Sayner and colleagues studied the *Pseudomonas aeruginosa* exotoxin Y (ExoY) and a novel chimeric soluble adenylyl cyclase (sACI/II) that was not active until it was bound by forskolin. When activated, both ExoY and sACI/II increased cAMP within the endothelial cell cytosol, an effect that led to inter-endothelial cell gap formation and increased permeability. Subsequent studies by Prasain et al. utilizing the sACI/II enzyme revealed that production of cAMP within the cytosolic compartments caused endothelial Tau phosphorylation and concomitant microtubule breakdown without inducing actin stress fiber formation. This collection of work identified microtubules as key targets of soluble adenylyl cyclases.

Just recently Ochoa and colleagues [20] discovered that ExoY has mixed cyclase activity, possessing the ability to increase cAMP and cGMP in endothelium. Both of these cyclic nucleotides are capable of inducing Tau hyperphosphorylation, but Tau appears to be most sensitive to the cAMP signal. Hyperphoshorylated Tau becomes insoluble to detergent extraction, an effect that is seen in tauopathies associated with chronic neurodegenerative diseases [21], [22]. This evidence indicates that ExoY causes an endothelial tauopathy. Other bacteria, including *Bacillus anthracis* [23] and *Bordetella pertussis* [24]–[26] utilize soluble cyclases as toxins, suggesting these bacteria may share a common pathogenic mechanism, that being the hyperphosphorylation and insolubility of Tau or Tau-like proteins.

Tau hyperphosphorylation prevents its binding to microtubules resulting in microtubule breakdown [27]. However, microtubules are dynamic structures, continually changing their organization and shape. Microtubule growth occurs at the GTP cap of microtubules; shortening occurs upon loss of the GTP cap [28]. Loss of the GTP cap leads to rapid microtubule shortening through a process termed a ‘catastrophe’ event. Following a catastrophe, the rate of reassembly is a critical determinant of microtubule length. Recurrent catastrophes shorten microtubules leading to their collective disassembly. It is not presently known whether ExoY and soluble ACs increase the rate of disassembly, as in a catastrophe event, or whether they decrease the rate of reassembly following a catastrophe. Here, we analyze microtubule assembly/disassembly characteristics and centrosome activity to test the hypothesis that ExoY activity disrupts microtubule dynamics in endothelial cells.

## Methods

### Cell Culture

Rat pulmonary microvascular endothelial cells (PMVECs) were isolated, characterized, and maintained using methods that have been reported previously [29]. Animal procedures were in accordance to NIH Guidelines and procedures were reviewed and approved by the University of South Alabama Institutional Animal Care and Use Committee (protocol 278237). Stable PMVEC transfectants over-expressing hTau S214A were detailed previously [30].

### Growth of *P. aeruginosa*

Two different strains of *P. aeruginosa* obtained from Dr. Dara W. Frank (Medical College of Wisconsin) were used for these studies [31]. One strain was a wild-type strain that contained an intact type III secretion system and produced native ExoY (*P. aeruginosa* ExoY^+^) while the other strain contained a fully functional type III secretion system but produced non-functional ExoY that was mutated at amino acid 81 (*P. aeruginosa* ExoY^K81M^). *P. aeruginosa* were grown on solid Vogel-Bonner minimal agar containing 400 µg/ml carbenicillin. For infection of PMVECs, cells were scraped into Kreb’s buffer and diluted to an MOI of 20. Ca^2+^ was added to 2 mM, media were removed from PMVECs, and the diluted bacteria were added to the cells for at least 3 hours to allow intoxication of either ExoY^+^ or ExoY^K81M^ to PMVECs [18].

### Immunofluorescence Microscopy

Labeling of cells with anti-alpha-tubulin antibodies (Sigma-Aldrich, St. Louis, MO) was performed as detailed previously [32]. To analyze effects of ExoY and ExoY^K81M^ on microtubule disassembly, PMVECs grown on coverslips were infected with *P. aeruginosa* as outlined above, and then the coverslips were placed at 0°C to induce microtubule disassembly. Individual coverslips were collected at either 0, 1, 2, or 3 minutes post-transfer to cold temperature, fixed in −20°C MeOH for 6–8 minutes, and labeled with antitubulin monoclonal antibodies (Sigma-Aldrich, St. Louis, MO) using methods described previously [32]. To assay effects of ExoY and ExoY^K81M^ on microtubule assembly, cells on coverslips were infected as detailed previously, and then at 3 hours post-infection the cells were placed on ice to cause microtubule disassembly. After complete breakdown of microtubules, the coverslips were transferred to a 37°C incubator to initiate microtubule re-growth and individual coverslips were collected at 0, 4, 8, and 12 minutes post-transfer to 37°C, fixed in −20°C MeOH, and then labeled with antitubulin antibodies. All experiments were repeated at least three times.

Tau immunolocalization was performed using the Rabbit-1 anti-Tau antibody (a generous gift from Dr. L.I. Binder, Northwestern University). For these experiments, cultured PMVECs were intoxicated with either ExoY^+^ or ExoY^K81M^ as detailed previously and then the cells were fixed using the crosslinking reagent ethylene glycol bis(succinimidylsuccinate) using procedures that were reported previously [34]. Following rinsing, the cells were blocked with glycine and then incubated with the Rabbit-1 antibody at a dilution of 1∶500. For some studies, tau and tubulin were co-localized in cells. For these experiments double-labeling using tau and tubulin antibodies was performed, images were collected at the same focal plane in the fluorescein and rhodamine channels, and the images were merged.

### Growth of Microtubules from Centrosomes Using Permeabilized Cells

Cells were infected with *P. aeruginosa* expressing either ExoY or ExoY^K81M^ and returned to the incubator for 3 hours. The cells were then placed on ice to disassemble cytoplasmic microtubules completely, and were permeabilized with a buffer composed of 80 mM PIPES, pH 6.8, 1 mM EGTA, 1 mM MgCl_2_ (PEM buffer) supplemented with 0.1% Triton X-100. Following rinsing with PEM buffer to remove all endogenous microtubule proteins and soluble cytosolic proteins, phosphocellulose-purified rat brain tubulin (diluted in PEM buffer containing 0.5 mM GTP) was added to the permeabilized cells, and the preparation was placed at 37°C for 15 minutes. Microtubules were fixed by the addition of glutaraldehyde to a final concentration of 2%, and then the coverslips were processed for antitubulin immunofluorescence using previously reported procedures [33]. Tubulin was isolated from rat brains using standard procedures [34].

### Immunoblot Analysis

To quantify the levels of assembled microtubules in cells, cells were infected with *P. aeruginosa* expressing either ExoY^+^ or ExoY^K81M^ as described above and then the cells were placed on ice to disassemble microtubules. The cells were then transferred to a 37°C incubator, and individual 35 mm dishes of cells were assayed at different times following transfer to 37°C to determine relative levels of tubulin monomer and polymer present in the cells using reported procedures [35]. To achieve this, the dishes of cells were rapidly rinsed with PBS and the buffer was removed. 100 µl of PEM buffer containing 0.1% Triton X-100 and either 25% glycerol or 0.1 ng/ml taxol was added to each dish to permeabilize the cells and release tubulin monomers from the cells. After 3 minutes the extraction buffer was removed and the cells were rinsed with an additional 50 µl of buffer. The rinse buffer was collected and pooled with the initial extraction solution, and then 150 µl of 2× SDS-PAGE sample buffer was added to the pooled solution containing the soluble monomeric tubulin. 300 µl of 1× SDS-PAGE sample buffer then was added to the residual cell ghosts containing assembled microtubules. The samples were boiled and equal amounts were loaded onto polyacrylamide gels. The proteins were resolved, transferred to nitrocellulose, and the blots were then probed using monoclonal anti-alpha-tubulin followed by peroxidase-labeled antimouse IgG antibody. The blots were developed using chemiluminescence procedures [19], [32], and the blots were scanned. The microtubule polymer to soluble tubulin ratio was measured, and the means ± S.E. were calculated. To measure levels of total Tau in cells, immunoblots were probed with the polyclonal Rabbit-1 anti-Tau antibody.

All immunoblot studies were performed four or five times, blots were scanned, and mean band intensities were calculated ± S.E. Means were analyzed using ANOVA, and differences were considered significant when the P value was <0.05.

### Tau Co-Pelleting with Microtubules

To assay whether endothelial tau would co-pellet with microtubules, microtubule pellets were analyzed by immunoblot analysis using the polyclonal anti-Tau antibody. For these experiments, PMVECs were either left untreated or were intoxicated with ExoY^+^ or ExoY^K81M^ as described previously. The cells were then homogenized in PEM buffer supplemented with 0.1 mM GTP, phosphatase inhibitors (Boston BioProducts #BP-479) and 1 µg/ml each of the protease inhibitors chymostatin, leupeptin, antipain, and pepstatin, and the homogenates were centrifuged at 100,000×g at 4°C for 2 hr. The supernatants were collected and then made 0.5 mM with GTP and 0.5 µM with taxol, and then they were incubated at 37°C for 30 mins to assemble microtubules. The extracts then were overlaid on a cushion of PEM buffer supplemented with 25% glycerol, GTP, taxol, phosphatase inhibitors, and protease inhibitors. The assembled microtubules were collected by centrifugation at 100,000×g for 40 mins at 30°C, and the pelleted microtubules were analyzed for the presence of tau using the Rabbit-1 antibody.

## Results

### ExoY causes the Breakdown of Microtubules

**Figure 1:**
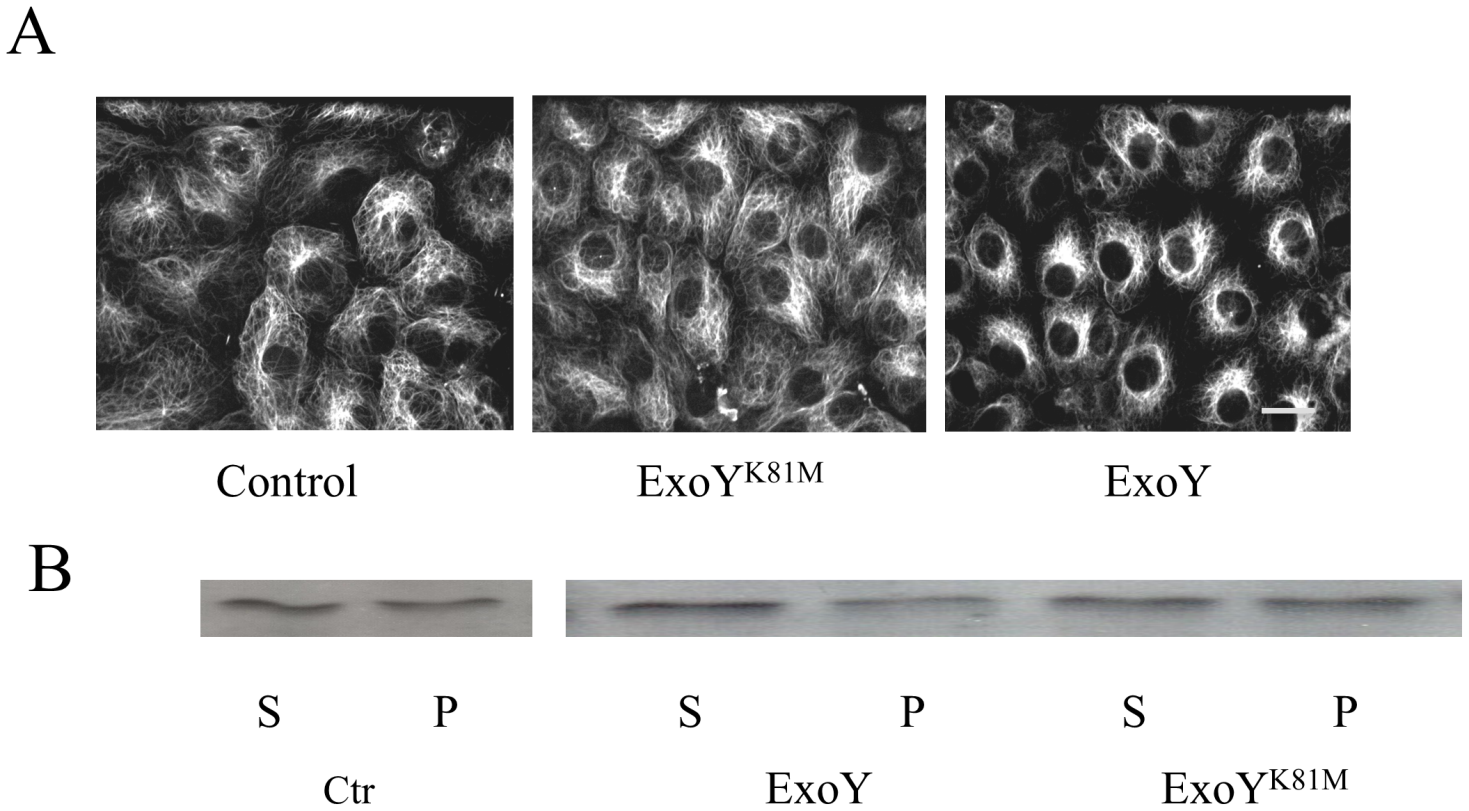
ExoY activity causes a decrease in microtubules in PMVECs. [**A**] PMVECs infected with *P.aeruginosa* expressing either non-functional K81M mutant ExoY (ExoY^K81M^; center) or wild type ExoY (right) were observed following processing for anti-tubulin immunofluorescence microscopy. Uninfected cells are also shown (left). Bar = 10 µm. [**B**] Levels of polymerized tubulin (P) and unpolymerized soluble tubulin (S) were quantified by immunoblot analysis using antibody against α-tubulin. Extracts obtained from untreated cells (Ctr) and from PMVECs infected with *P. aeruginosa* expressing either ExoY^K81M^ or ExoY^+^ are shown. The ratio of polymerized tubulin to soluble tubulin was significantly less in cells containing wild type ExoY^+^ [0.29±0.06 *vs.* 0.44±0.05 (ExoY^K81M^) and 0.49±0.08 (Ctr); n = 5; P<0.05 compared to both ExoY^K81M^ and untreated control].

Following infection of PMVECs with *P. aeruginosa* ExoY^+^, cell adhesion processes were disrupted and gaps formed between adjacent cells [7], [18]. To determine whether microtubule cytoskeletal alterations could be contributing to the formation of endothelial gaps, cells infected with *P. aeruginosa* ExoY^+^ or *P. aeruginosa* ExoY^K81M^ were analyzed using anti-tubulin immunofluorescence microscopy. As shown in Figure 1, obvious differences in the microtubule cytoskeleton were noted. Specifically, fewer microtubules were observed in cells infected with *P. aeruginosa* ExoY^+^, and microtubules were rarely observed extending completely to the cell cortex. In contrast, no obvious effects on microtubules were detected in cells infected with *P. aeruginosa* ExoY^K81M^ (Figure 1). The apparent decrease in the number of microtubules in *P. aeruginosa* ExoY^+^-infected cells was verified by immunoblot analysis using anti-tubulin antibodies (Figure 1) which demonstrated less microtubule polymer in *P. aeruginosa* ExoY^+^-infected cells relative to the amount of tubulin polymer in uninfected control cells or cells infected with *P. aeruginosa* ExoY^K81M^ [0.29±0.06 *vs.* 0.44±0.05 (ExoY^K81M^) and 0.49±0.08 (Ctr); n = 5; P<0.05 compared to both ExoY^K81M^ and untreated control].

### ExoY Disrupts Microtubule Assembly

**Figure 2:**
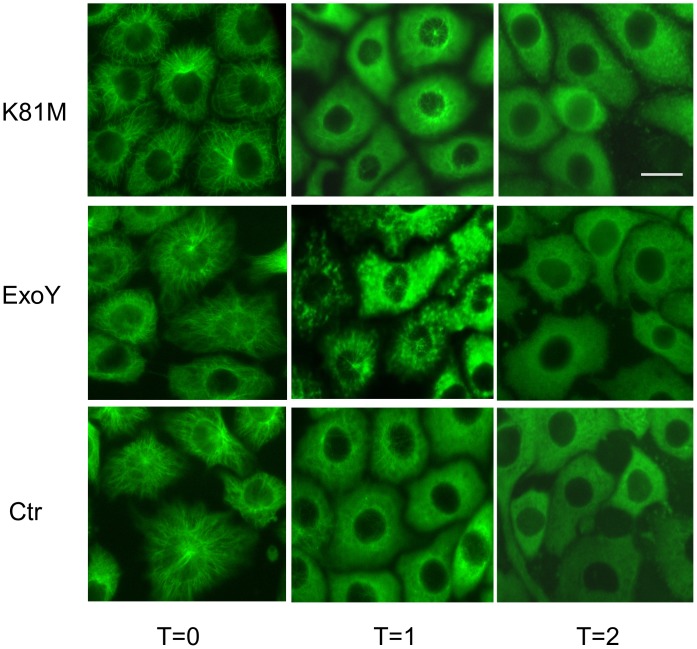
ExoY activity does not noticeably affect microtubule disassembly. PMVECs were infected with *P. aeruginosa* expressing either ExoY^K81M^ (upper panels) or wild type ExoY (middle panels). Following infection, cells were either fixed before microtubule disassembly was induced (left) or at 1 (middle) or 2 minutes (right) after being placed at 0°C. As shown, disassembly was complete in both control (Ctr), ExoY^K81M^ and ExoY^+^ expressing cells by 2 minutes after transfer to 0°C. Bar = 10 µm.

The decrease in microtubules induced by *P. aeruginosa* ExoY^+^ infection could occur as a result of changes in either microtubule assembly or disassembly behavior. Each of these possibilities was investigated individually. Initially, studies were performed to assess microtubule disassembly in infected cells. For these studies, cells were infected with *P. aeruginosa* ExoY^+^ or *P. aeruginosa* ExoY^K81M^ for 3 hours. After infection, the rates of microtubule disassembly were determined. To induce disassembly, the Petri dishes were placed on ice and individual coverslips were collected at either 0, 1, 2, or 3 minutes after chilling. The cells were then processed for anti-tubulin immunofluorescence microscopy. As shown in Figure 2, no obvious difference was detected in microtubule disassembly in PMVECs infected with either *P. aeruginosa* ExoY^+^ or *P. aeruginosa* ExoY^K81M^, with complete microtubule breakdown detected in uninfected control and both populations of infected cell types within two minutes.

**Figure 3:**
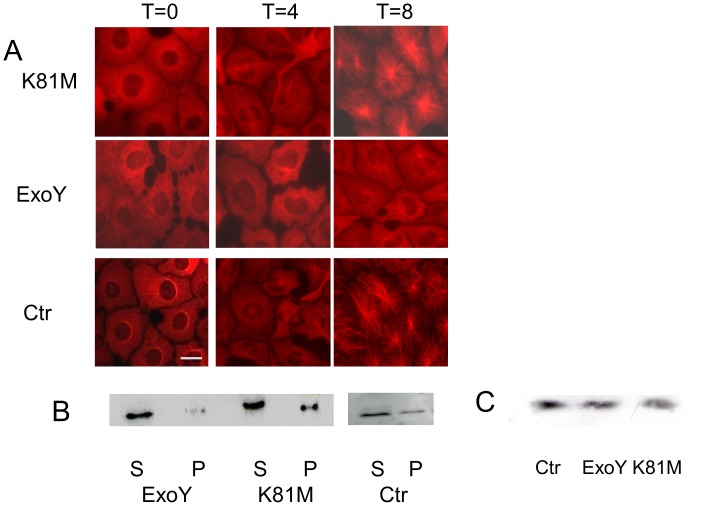
ExoY activity affects microtubule assembly in PMVECs. PMVECs were infected with *P. aeruginosa* expressing either ExoY^K81M^ or ExoY^+^. Untreated control cells (Ctr) and infected cells were then placed on ice to induce microtubule disassembly. Microtubule re-growth was initiated by transferring the cells to 37°C. [**A.**] Individual coverslips were fixed either at the time of transfer (T = 0) or at varying times after transfer to 37°C. The coverslips then were labeled with antitubulin antibodies. Cells at 4 and 8 minutes post-transfer are shown; microtubule growth is apparent by 4 minutes and peripheral microtubules are resolved by 8 minutes in untreated and K81M infected cells. Microtubule re-growth lagged significantly in cells intoxicated with wt ExoY. Bar = 10 µm. [**B.**] Polymerized (P) and soluble unpolymerized (S) tubulin levels were quantified by immunoblot analysis using antitubulin antibodies. To obtain soluble and polymer fractions, control cells and cells infected with bacteria expressing either ExoY^K81M^ or ExoY^+^ were extracted at 8 minutes post-transfer to 37°C. The ratio of polymerized tubulin to soluble tubulin was significantly less in cells containing wild type ExoY [0.10±0.06 *vs.* 0.36±0.10 (K81M) and 0.41±0.09 (Ctr); n = 4; P<0.05 compared to both K81M and untreated cells]. [**C.**] Tau levels are unchanged following cold treatment to disassemble microtubules. Cells were treated with cold and then whole extracts were collected from control cells (Ctr) and from cells that were intoxicated with either ExoY^+^ or ExoY^K81M^. The extracts were then probed for tau levels using polyclonal anti-tau antibody.

Similar types of immunofluorescence studies were performed to assess whether ExoY affected microtubule assembly behavior following infection. To assay assembly, cells were placed on ice initially to disassemble microtubules completely, and then were transferred to 37°C to initiate microtubule re-assembly. Individual coverslips were collected at 0, 4, 8, and 12 minutes after warming, fixed, and processed for anti-tubulin immunofluorescence microscopy. In contrast to what was observed when assessing microtubule disassembly, microtubule assembly was depressed in cells infected with *P. aeruginosa* ExoY^+^ when compared to control cells and cells infected with *P. aeruginosa* ExoY^K81M^ (Figure 3). These results were verified by immunoblot analysis (Figure 3), which demonstrated reduced levels of microtubule polymer in cells infected with ExoY-expressing bacteria relative to levels in control and ExoY^K81M^ intoxicated cells [0.10±0.06 *vs.* 0.36±0.10 (K81M) and 0.41±0.09 (Ctr); n = 4; P<0.05 compared to both K81M and untreated cells]. Importantly, tau levels did not change during the cold treatment in any of the cell populations (Figure 3). These results demonstrate that the decreased numbers of microtubules observed in PMVECs following infection with *P. aeruginosa* occur, at least in part, as a result of decreased capacity to assemble microtubules.

### ExoY does not Impair Centrosome Nucleation

**Figure 4:**
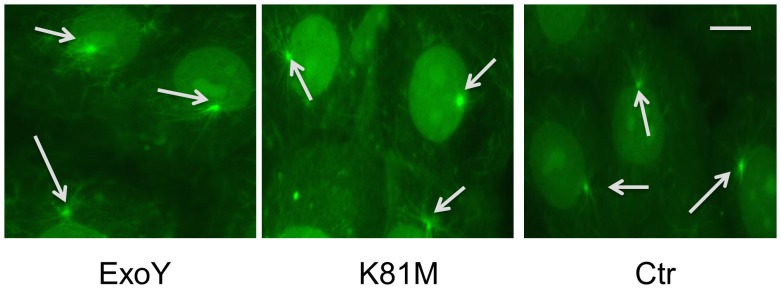
ExoY activity does not noticeably affect microtubule assembly from PMVEC centrosomes. PMVECs were infected with *P. aeruginosa* expressing either ExoY^K81M^ (middle panel) or ExoY^+^ (left panel). Untreated cells (Ctr) and infected cells were extracted to remove soluble proteins and then purified rat brain tubulin was added and microtubule nucleation was initiated by incubating at 37°C for 15 min. The preparations then were fixed and labeled with antitubulin antibodies. Centrosome nucleation of microtubules is shown by arrows. Bar = 10 µm.

The decrease in microtubule nucleation observed in Figure 3 could be due either to effects on the centrosome or to effects on microtubule-associated proteins that modulate microtubule behavior. Studies were performed to assess each of these possibilities. Initially, the microtubule nucleating capacity of centrosomes in PMVECs was analyzed following infection with *P. aeruginosa* ExoY^+^. For these studies, cells were infected with bacteria, incubated for three hours to permit transfer of ExoY to infected PMVECs, and then placed on ice to disassemble cellular microtubules. The cells then were permeabilized to remove endogenous microtubule proteins and other soluble cellular proteins. Following rinsing, pure tubulin was added and the preparations were warmed to 37°C to induce microtubule assembly. The extracted cells then were fixed and observed. As shown in Figure 4, no difference in the ability of centrosomes to initiate microtubule nucleation could be detected when comparing cells that had been infected with *P. aeruginosa* ExoY^+^ to control cells and to cells that had been infected with *P. aeruginosa* ExoY^K81M^, suggesting that the cyclic nucleotides being produced by ExoY were not targeting the microtubule nucleating sites within the centrosomes at the early time points tested post-infection.

### ExoY Induces Tau Ser-214 Phosphorylation Necessary to Impair Microtubule Reassembly

**Figure 5:**
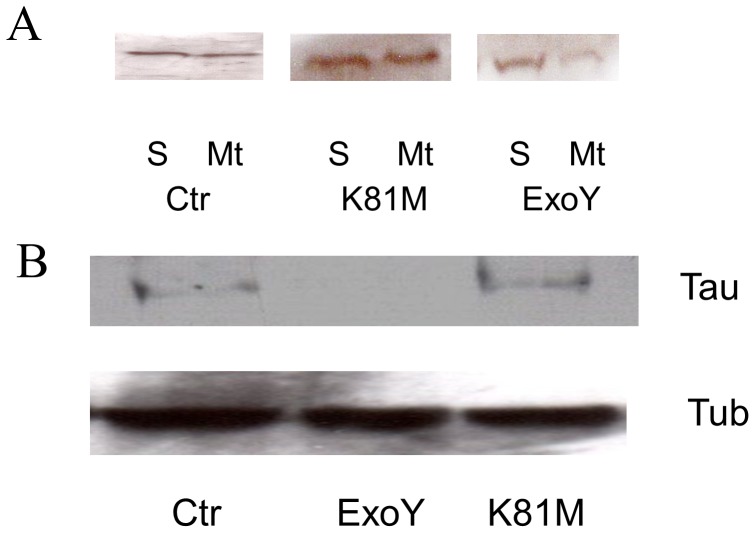
Phosphorylation causes Tau to dissociate from microtubules. [**A.**] Untreated cells (Ctr) as well as cells that were infected with P. aeruginosa expressing either ExoY^K81M^ or ExoY^+^ were incubated, and then extracts were prepared and soluble fractions and cell ghosts containing intact microtubules were collected. Both microtubule-associated (Mt) and soluble free Tau (S) were assayed by immunoblot using a pan-Tau antibody. Significantly less tau was present in cells ghosts prepared from cells intoxicated with ExoY^+^ compared to those prepared from either untreated or K81M-intoxicated cells [0.22±0.09 *vs.* 0.44±0.08 (Ctr) and 0.46±0.04 (K81M); n = 4; P<0.05 compared to both K81M and untreated cells]. [**B.**] **Tau co-pellets with taxol-stabilized microtubules.** Extracts were prepared from untreated control PMVECs (Ctr) and from cells infected with bacteria expressing either ExoY^+^ (middle) or ExoY^K81M^ (right), microtubules were assembled by addition of taxol, and then the assembled microtubules were pelleted through a sucrose cushion. The pelleted microtubules were then probed by immunoblot using polyclonal anti-tau antibody (top). The blot was then stripped and re-probed using antitubulin antibody (bottom).

Previous studies demonstrated that increased levels of cyclic nucleotides in the cytosol of endothelial cells can lead to phosphorylation of the microtubule-associated protein Tau on Ser-214 [14], [17], [19], [20]. Since Tau hyperphosphorylation causes dissociation of Tau from microtubules in neuronal cells, studies were performed to test whether hyperphosphorylation of endothelial Tau by *P. aeruginosa* ExoY^+^ -infected cells caused dissociation of Tau from endothelial microtubules. As shown in Figure 5, immunoblot analysis demonstrated that Tau dissociated from microtubules following infection of endothelial cells with *P. aeruginosa* ExoY^+^, with significantly less tau bound to microtubules in ExoY intoxicated cells relative to the amount bound in control and K81M treated cells [0.22±0.09 *vs.* 0.44±0.08 (Ctr) and 0.46±0.04 (K81M); n = 4; P<0.05 compared to both K81M and untreated cells]. To further investigate the effects of tau phosphorylation on its ability to bind microtubules, lysates were collected from control and ExoY^+^ infected PMVECs and microtubules were assembled in the extracts by the addition of taxol. Following centrifugation, the pelleted microtubules were assayed by immunoblot using polyclonal anti-Tau antibody. As shown in Figure 5, phosphorylated tau was unable to co-pellet with microtubules assembled from PMVEC extracts while tau in control extracts and extracts prepared from cells infected with ExoY^K81M^ co-pelleted.

**Figure 6:**
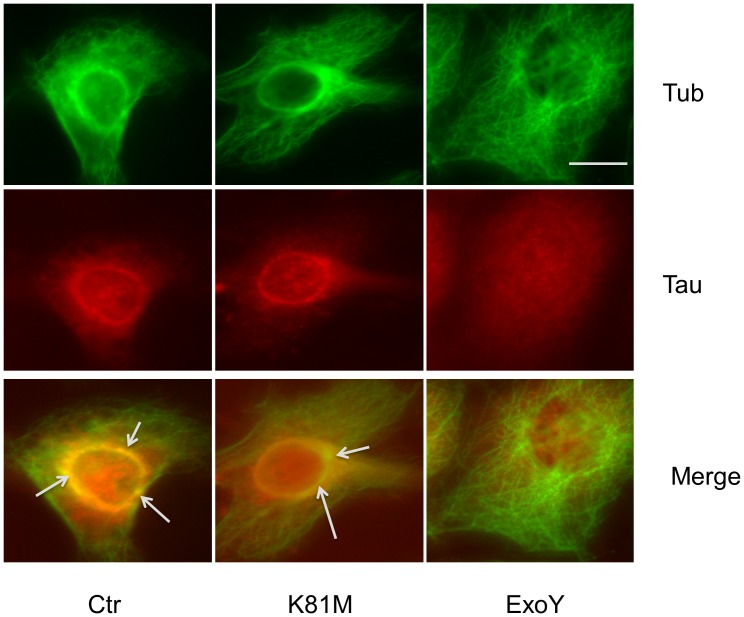
ExoY intoxication disrupts Tau localization. Tau localization in control PMVECs (Ctr) and cells intoxicated with bacteria producing either ExoY^K81M^ or wild-type ExoY^+^. The cells then were fixed and processed for immunofluorescence using both tubulin (green) and tau (red) antibodies. Images were collected at the same focal plane, the images were merged and co-localization of tubulin and tau can be seen (arrows) in the control and ExoY^K81M^ infected cells. No co-localization was observed in cells intoxicated with ExoY^+^. Bar = 10 µm.

To assay the effects of phosphorylation on tau localization, immunofluorescence microscopy was performed using ExoY^+^ and ExoY^K81M^ infected endothelial cells. These studies demonstrated tau arranged in a punctate manner in control cells and in cells infected with ExoY^K81M^ (Figure 6). Moreover, tau immunoreactivity appeared to be enriched near the nucleus, presumably in the region of the centrosome where microtubule density is greatest. To confirm these possibilities, double labeling for tubulin and tau was performed and the images were merged. As shown in Figure 6, colocalization of tubulin and tau could be observed in regions where microtubule density was greatest in control and ExoY^K81M^ infected cells. In contrast, tau was randomly dispersed in the cytoplasm of cells infected with ExoY^+^ (Figure 6).

**Figure 7:**
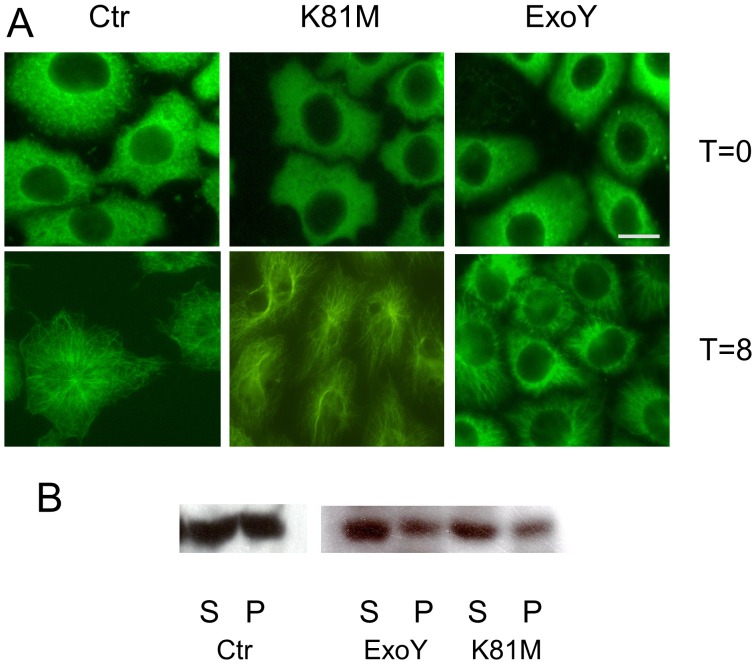
Phosphorylation of tau is essential for disrupted microtubule assembly. Over-expression of a non-phosphorylatable form of Tau (S214A mutant) rescues PMVECs from effects of ExoY^+^ on microtubule assembly. PMVECs were stably transfected with a cDNA encoding a form of Tau mutated at the PKA phosphorylation site and then infected with *P. aeruginosa* encoding either ExoY^K81M^ or ExoY^+^. Microtubules were disassembled by incubation on ice, and then microtubule re-assembly was initiated by transferring cells to 37°C. [**A.**] S214A Tau-expressing control cells (Ctr) and cells infected with either ExoY^K81M^ or ExoY^+^ were fixed and labeled with antitubulin antibodies either at the time of transfer to 37°C (top) or at 8 minutes post-transfer (bottom). Bar = 10 µm. [**B.**] Polymerized (P) and soluble unpolymerized (S) tubulin levels were quantified in S214A-expressing cells at 8 minutes post-transfer to 37°C. Extracts were prepared from control cells (Ctr) and from cells infected with *P. aeruginosa* expressing either ExoY^K81M^ or ExoY^+^. There was no significant difference in the ratio of microtubule polymer to soluble tubulin when cells infected with bacteria expressing ExoY^+^ were compared to those expressing ExoY^K81M^ or uninfected control cells [0.35±0.8 *vs.* 0.38±0.06 (K81M) and 0.44±0.11 (Ctr); n = 5].

To investigate whether hyperphosphorylation of Tau was responsible for the change in microtubule assembly behavior identified following PMVECs infection with *P. aeruginosa* ExoY^+^, the previously described microtubule disassembly and reassembly studies were repeated using PMVECs stably transfected with a mutant form of human Tau (hTau) that could not be phosphorylated at Ser-214 (S214A). In cells expressing the non-phosphorylatable S214A mutant hTau, ExoY had no effects on microtubule assembly (Figure 7) and disassembly (not shown) following infection. Moreover, immunoblot analysis using anti-tubulin antibody confirmed the immunofluorescence observations by demonstrating equivalent ratios of microtubule polymer to tubulin monomer in untreated control, ExoY^+^- and ExoY^K81M^ -infected PMVECs [Figure 7; 0.35±0.8 *vs.* 0.38±0.06 (K81M) and 0.44±0.11 (Control); n = 5]. These results demonstrate that expression of the non-phosphorylatable form of hTau rescued the microtubule cytoskeleton from effects due to ExoY activity.

## Discussion

*Pseudomonas aeruginosa* is an opportunistic bacterium that causes acute and chronic pulmonary infections. Characteristic of these infections is a fulminating pneumonia that includes edema, fluid in the airways, and migration of various types of inflammatory cells into the alveolar spaces [36]–[38]. These observations suggest a breakdown of the endothelial barrier following infection, and this possibility has been supported by *in vitro* experimental studies that demonstrated pulmonary endothelial barrier disruption following *Pseudomonas* infection [18]. The studies reported here demonstrate that microtubules are disrupted in PMVECs following *Pseudomonas* infection and further show that Tau phosphorylation results in decreased microtubule assembly, a putative cause of endothelial barrier disruption. That Tau is the principal target of toxin introduced by *Pseudomonas aeruginosa* is supported by studies in which cells types expressing non-phosphorylatable Tau were analyzed.

Studies by Rich and colleagues [11], [13], [39] demonstrated that cells maintain a cAMP gradient from the cortical membrane region to deeper cytosol areas, with the cortical pool of cAMP being 10-fold greater than levels in the bulk cytosol. This gradient of cAMP is essential for maintaining endothelial barrier strength [5], [7], [14], and cAMP produced near the plasma membrane achieves this by stabilizing the cortical actin rim, which is involved in maintaining junctional adhesion complexes. A similar concept has been advanced to explain how membrane and soluble guanylyl cyclases, in response to atrial natriuretic peptide and nitric oxide, respectively, distinctly regulate the endothelial cell barrier [40]. At present, relatively little is known about how cGMP signals are compartmentalized within endothelium. Regardless, microtubules extending toward the cell cortex interact with actin filaments and assist with maintaining cell shape and barrier integrity; treatments leading to microtubule breakdown result in cell retraction and barrier disruption [32], [41]–[43]. In this paper, data demonstrate that the *P. aeruginosa* toxin ExoY, which is an adenylyl and guanylyl cyclase molecule lacking membrane anchoring domains, decreased levels of microtubules in PMVECs and induced barrier breakdown by generating cyclic nucleotides within a cytosolic domain. Investigations were performed to identify potential targets of this cytosolic pool of cAMP and cGMP, and individual studies assayed effects on the activity of the centrosomes and the microtubule-associated protein Tau. The centrosome is the principal microtubule-nucleating site in cells, and multiple A-kinase anchoring proteins are localized within the centrosome complex [44], [45]. Studies outlined here directly tested whether cAMP and/or cGMP produced by ExoY modulated the microtubule nucleating capacity of centrosomes, and no apparent change in microtubule nucleating activity was detected at the time points tested. These observations indicate that the microtubule nucleating sites localized within the centrosome complex most likely are not targeted directly to lead to microtubule cytoskeletal disruption following *P. aeruginosa* infection, at least during the early stages of cellular response.

Microtubules are dynamic polymers capable of rapid assembly and disassembly. In cells, rates of assembly and disassembly are modulated by microtubule-associated proteins and by direct post-translational modification of tubulin monomers [46], [47]. One of the major microtubule-associated proteins in mammalian neuronal cells is Tau. In neurons, Tau is responsible for bundling axonal microtubules, and abnormal Tau hyperphosphorylation and insolubility has been linked to formation of paired helical filaments in Alzheimer’s disease [48], [49] and to other neurological insufficiencies [50]–[52]. Previous studies have shown that Ser-214 of Tau is phosphorylated by cAMP-and/or cGMP-dependent kinases [30], [53], and phosphorylation of Tau results in dissociation of Tau from the microtubule lattice. The *in vivo* results reported here are in agreement with these previous *in vitro* observations and also demonstrate that phosphorylation of Tau on Ser-214 can be used by microvascular endothelial cells for modulating microtubule dynamics. Moreover, these data indicate the phosphorylation of Tau occurring as a result of ExoY activity contributes to the pulmonary pathologies associated with *Pseudomonas aeruginosa* infection by disrupting microtubule assembly processes in PMVECs resulting in barrier disruption.

Tau is classically considered to be a neuronal protein, although the presence of a Tau protein in non-neuronal mammalian cells has been described [54]–[57]. However, the function of Tau in non-neuronal cells has not been investigated in detail. The studies reported here demonstrate that Tau and the phosphorylation of Tau are mechanisms used by PMVECs to modulate microtubule dynamics and to regulate microtubule stability. Data demonstrate that PMVECs containing elevated levels of phosphor-Tau contain less tubulin polymer than those with lower levels of phosphorylated Tau, and the studies reported here further demonstrate that microtubule assembly is disrupted in cells containing phosphor-Tau. In contrast, effects on microtubule disassembly by Tau phosphorylation were not detected in these studies. One possible explanation is that the methods used were not sensitive enough to detect effects on disassembly behavior. Alternatively, it has been demonstrated by Panda et al. [58] that isoforms of Tau differentially modulate microtubule kinetics, with some forms of Tau being able to stabilize microtubules against disassembly while other isoforms have little effect on microtubule disassembly behavior. Studies using either Tau purified from PMVECs or real time imaging using fluorescent tubulin will be necessary to determine whether this non-neuronal Tau modulates microtubule disassembly behavior.

Our studies have not determined whether it is the cAMP or cGMP signal, or both, that is responsible for decreasing microtubule assembly. Data presented here and the work of Ochoa et al. [20] suggests Tau hyperphosphorylation and insolubility is responsible for this observation. While Tau hyperphosphorylation is necessary to inhibit microtubule assembly, we have not ruled out the possibility that ExoY catalytic activity itself also impairs microtubule assembly. Microtubules grow at their GTP cap. Since ExoY possesses guanylyl cyclase activity, it may consume GTP in competing with microtubules for GTP binding. Kinetic analysis of ExoY enzyme activity will be necessary to better address this possibility.

In summary, *P. aeruginosa* and other types of bacteria induce pulmonary endothelial barrier breakdown following infection. Pseudomonas uses a type III secretion system to transfer a promiscuous cyclase, ExoY, to the cytoplasm of pulmonary microvascular endothelial cells during infection processes, and data presented here demonstrate that the microtubule associated protein Tau is the target of cyclic nucleotides produced following infection. Phosphorylation of Tau disrupts microtubules in PMVECs leading to barrier disruption contributing to the pathologies associated with *Pseudomonas* infections. Identification of molecular targets following *Pseudomonas* infection could lead to more rational treatments for these types of infections.
